# Durable wall lining for malaria control in Liberia: results of a cluster randomized trial

**DOI:** 10.1186/s12936-022-04429-7

**Published:** 2023-01-12

**Authors:** David Giesbrecht, Tuwuyor G. Belleh, Julie Pontarollo, Victor S. Hinneh, Oliver Pratt, Sajid Kamal, Richard Allan

**Affiliations:** 1 Department of Pathology and Laboratory Medicine, Brown University, Providence, RI USA; 2 ABT Associates, Monrovia, Liberia; 3 Liberia National Malaria Control Programme, Monrovia, Liberia; 4 The MENTOR Initiative, Haywards Heath, UK

**Keywords:** Malaria, Vectors, LLINs, Durable wall lining, Rural housing

## Abstract

**Background:**

Malaria control in Liberia depends upon universal coverage with pyrethroid-impregnated long-lasting insecticidal nets (LLINs). Despite regular mass distribution, LLIN coverage and usage is patchy. Pyrethroid resistance in malaria vectors may further reduce LLIN efficacy. Durable Wall Lining (DWL), a novel material treated with two non-pyrethroid class insecticides, was designed to be installed onto the surface of inner walls, and cover openings and ceiling surfaces of rural houses.

**Objectives:**

**Aim:**

To determine the malaria control efficacy of DWL.

**Primary objective:**

To determine if DWL has an additional protective effect in an area of pyrethroid resistance.

**Secondary objectives:**

To compare surface bio-availability of insecticides and entomological effectiveness over the study duration.

**Design:**

A cluster randomized trial.

**Participants:**

Children aged 2–59 months.

**Control arm:**

50 houses per 20 clusters, all of which received LLIN within the previous 12 months.

**Active arm:**

50 houses per 20 experimental clusters, all of which received LLINs with the previous 12 months, and had internal walls and ceilings lined with DWL.

**Randomisation:**

Cluster villages were randomly allocated to control or active arms, and paired on 4 covariates.

**Main outcome measures:**

**Primary measure:**

Prevalence of infection with *P. falciparum* in children aged 2 to 59 months.

**Secondary measure:**

Surface bioavailability and entomological effectiveness of DWL active ingredients.

**Results:**

*Plasmodium falciparum* prevalence in active clusters after 12 months was 34.6% compared to 40.1% in control clusters (p = 0.052). The effect varied with elevation and was significant (RR = 1.3, p = 0.022) in 14 pairs of upland villages. It was not significant (RR = 1.3, p = 0.344) in 6 pairs of coastal villages. Pooled risk ratio (RR) was calculated in SAS (Cary, NC, USA) using the Cochran–Mantel–Haenszel (CMH) test for upland and coastal cluster pairs. DWL efficacy was sustained at almost 100% for 12 months.

**Conclusions:**

Findings indicate that DWL is a scalable and effective malaria control intervention in stable transmission areas with pyrethroid-resistant vectors, where LLIN usage is difficult to achieve, and where local housing designs include large gable and eve openings.

*Trial registration* ClinicalTrials.gov identifier: NCT02448745 (19 May 2015): https://clinicaltrials.gov/ct2/show/NCT02448745

**Supplementary Information:**

The online version contains supplementary material available at 10.1186/s12936-022-04429-7.

## Background

Controlling malaria vectors in sub-Saharan Africa is a daunting task, and is now made more difficult with the development of pyrethroid resistance. The slow pace of bringing new insecticides to market requires new thinking around insecticide delivery to target indoor-biting *Anopheles* mosquitoes. One such proposal is the use of durable, insecticidal wall liners (DWL) [1]. DWL could provide several advantages over long-lasting insecticidal nets (LLINs) and indoor residual spraying (IRS). Like IRS, DWL covers surfaces where mosquitoes rest, but insecticide-impregnated plastics overcome the problem of deterioration of indoor wall surfaces. Like LLINs, DWL can be deployed to intercept host-seeking mosquitoes, but is installed well away from sleeping children, thereby reducing their contact with the active ingredients. Currently, the evidence for DWL for malaria control is limited to assessments of the feasibility of installation at small scales as well as hut trials [1–6].

The forerunner of DWL, insecticide-treated plastic sheeting (ITPS), was first developed in emergency settings where insecticidal materials were needed for displaced persons [7, 8]. In this setting, LLIN usage is often hampered by lack of shelter, so providing shelter that doubled as a malaria control product was an attractive innovation. Early ITPS trials in Afghanistan [7, 9] and Sierra Leone [8] demonstrated efficacy against *Anopheles* mosquitoes. In the case of the Sierra Leone study, installation of ITPS was associated with a marked reduction in malaria incidence [8]. Subsequently, ITPS was tested in more politically stable settings with disappointing results. A trial of ITPS treated with a carbamate insecticide found no benefit over background LLIN usage [10]. Similarly, limited efficacy was observed in trials using ITPS treated with a pyrethroid [11], and no additional protection was observed when organophosphate treated ITPS was installed in houses, together with LLINs [6].

To extend the ITPS concept to permanent communities, insecticidal materials were developed to cover walls and reduce entry by mosquitoes while maintaining airflow. These materials are distinct from ITPS and are referred hereafter to as DWL. DWL can be deployed by homeowners in a variety of ways, and can be thought of as household improvement, a mosquito control approach with a longstanding body of research [12, 13]. Using DWL to prevent mosquito entry is a logical application of its properties, yet few studies have been conducted to test this idea, and much of the research to date has limited DWL installation to walls without covering eaves [3, 4]. Notably, the very first study of a rudimentary DWL in 1990 used insecticidal eave coverings made from locally available burlap, and this resulted in reduced indoor biting [12]. Another similar application of DWL in Mozambique tested 3 different DWL screening materials, which were shown to be effective in reducing indoor biting [14]. By covering eaves and gables, entry by host-seeking *Anopheles gambiae *sensu lato (*s.l.*) and *Anopheles funestus* was reduced. More recent research into the efficacy of covering eaves with DWL suffered from lack of consistency in installation and showed no effect [5]. Finally, a related approach is the use of eave tubes, where locally available bricks and plaster close the eaves, and insecticide-treated tubes are installed [15, 16]. This creates an area of focused human odours emanating from the house where host-seeking mosquitoes come in contact with insecticide. In Côte d’Ivoire, a cluster randomized trial of eave tubes resulted in a dramatic reduction in indoor biting and malaria cases [16].

Although the use of insecticidal materials as eave and wall coverings is not comprehensively studied, the existing data suggests that DWL deployed to intercept mosquitoes during entry through eaves and to cover walls will kill mosquitoes during both host-seeking and indoor resting phases of blood feeding. Mosquitoes attempting to enter a DWL-protected house may: (1.) fail to enter due to the mechanical barrier on the eaves; (2.) enter the house through openings, but receive a dose of insecticide during an exploratory period; or (3.) enter the house, bite an occupant and rest on the insecticidal wall covering, thereby receiving a lethal dose of insecticide. Covering indoor surfaces with insecticidal materials exploits resting behaviour following a blood meal; contact with insecticidal materials on walls and ceilings is likely in the hours following engorgement. DWL deployed as a wall covering and eave covering, with minimal places of entry, function similarly to LLINs by preventing biting and exposing mosquitoes to insecticides during host-seeking; the treated surfaces function like Indoor Residual Spraying (IRS) by placing insecticide on indoor surfaces where mosquitoes are likely to rest.

Before the current study was implemented, a deltamethrin-impregnated shade cloth DWL similar to that used in Mozambique [14] was found to be ineffective against pyrethroid-resistant mosquitoes in Liberia [17]. In response to this failure, a second prototype DWL product was developed using a combination of fenpyroximate and abamectin. Together, these 2 insecticides were expected to provide an effective approach against pyrethroid resistant *An. gambiae s.l.* When fenpyroximate abamectin DWL is provided in combination with pyrethroid LLINs, three insecticide classes are delivered to mosquitoes within the same house, potentially providing a means of shifting the age class of indoor biting malaria vectors, killing significant numbers of infectious mosquitoes, and thereby reducing malaria incidence.

### Aim

To determine the efficacy of DWL with novel insecticides as a malaria control measure in an area where malaria vectors are resistant to pyrethroids, and transmission is high to moderate.

### Primary objective

To determine if second generation DWL with a novel insecticide has an additional protective effect against malaria in an area where pyrethroid LLIN ownership is common, but pyrethroid resistance and poor usage rates hamper their efficacy. This will be measured by *P. falciparum* prevalence in under 5 s.

### Secondary objectives

To compare the surface bio-availability of two new active ingredients in DWL over the duration of the study.

## Methods

### Trial design

**Fig. 1:**
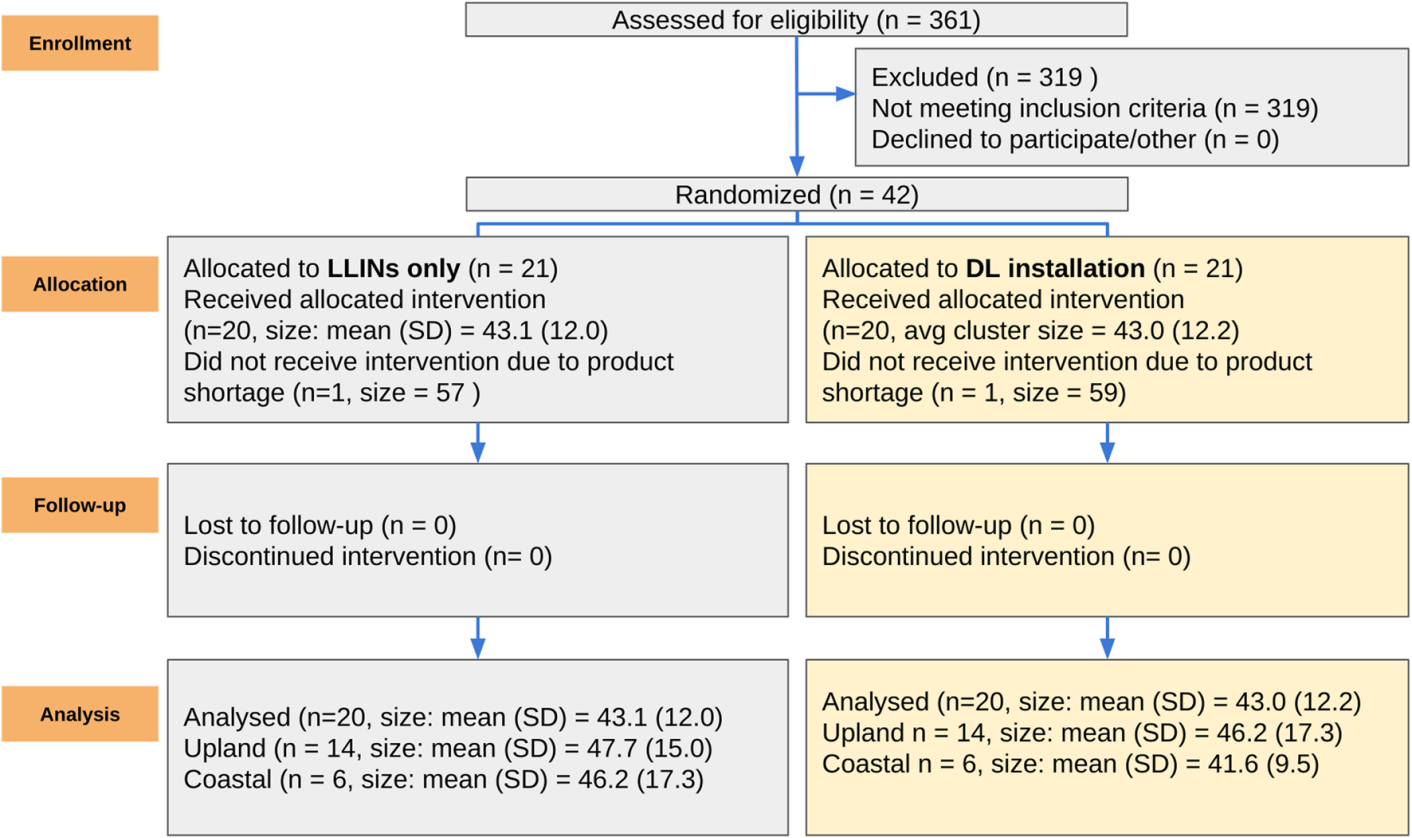
Flowchart of study design—Bomi County, Liberia. Initially, 42 clusters were identified, each housing approximately 50 children and with houses were spatially clustered. Participants were recruited in the 42 clusters for the baseline epidemiological survey. Two of the 42 clusters subsequently became insecure and had to be removed. The remaining 40 clusters were matched based on *P. falciparum* prevalence, population size, LLIN usage and district. Following random allocation to continued use of LLINs or use of LLINs plus DWL installation, 20 clusters were selected to receive DWL. Analysis was conducted for all 40 clusters, and also separately for 28 upland clusters, and for 12 coastal clusters, to determine any regional specific difference

To test the efficacy of DWL in a region with high malaria transmission throughout the year, a paired, randomized controlled trial was conducted in 40 clusters in Bomi County, Liberia (Fig. 1). Candidate clusters were identified from census data. The GPS data in each of the 3 districts was plotted as open circles at 1:80,000 and examined for relatively isolated villages or groups of villages. The number of occupants and their age was recorded. If villages had less than 60 under 5 s, the map was searched for nearby villages and these were added to form a cluster with 2 or more villages. Each of these villages was matched back to the raw data and the individuals under 5 years old were counted. If the village had between approximately 60 and 100 children under 5, it was selected as a cluster. The study census data was compared to a 1:90,000 Liberia Institute of Statistics and Geo-Information Services (LISGIS) map of Bomi villages and health centres. Where selected clusters were found on district or county borders, Google Maps satellite view was searched to look for major population centres on the other side of the border. If these were found, the cluster was excluded. Clusters less than 1 km away from another cluster or close to large population centres were excluded. Villages along main roads were generally avoided since settlements tend to be scattered along the road. A few villages along major roads were considered to be fairly discrete population centres and were included as clusters. From this census data, 42 clusters were identified that each housed approximately 50 children and where houses were spatially clustered. Participants were recruited in 42 clusters for the baseline epidemiological survey. Two of the 42 clusters proved to be subsequently insecure or non-accessible due to the early stages of an Ebola outbreak, and were removed.

### Participants

All children aged 2 months to 59 months, with consenting parents, living in the selected clusters (villages) spread across upland and coastal areas of Bomi County, were deemed eligible for inclusion.

### Intervention

**Fig. 2:**
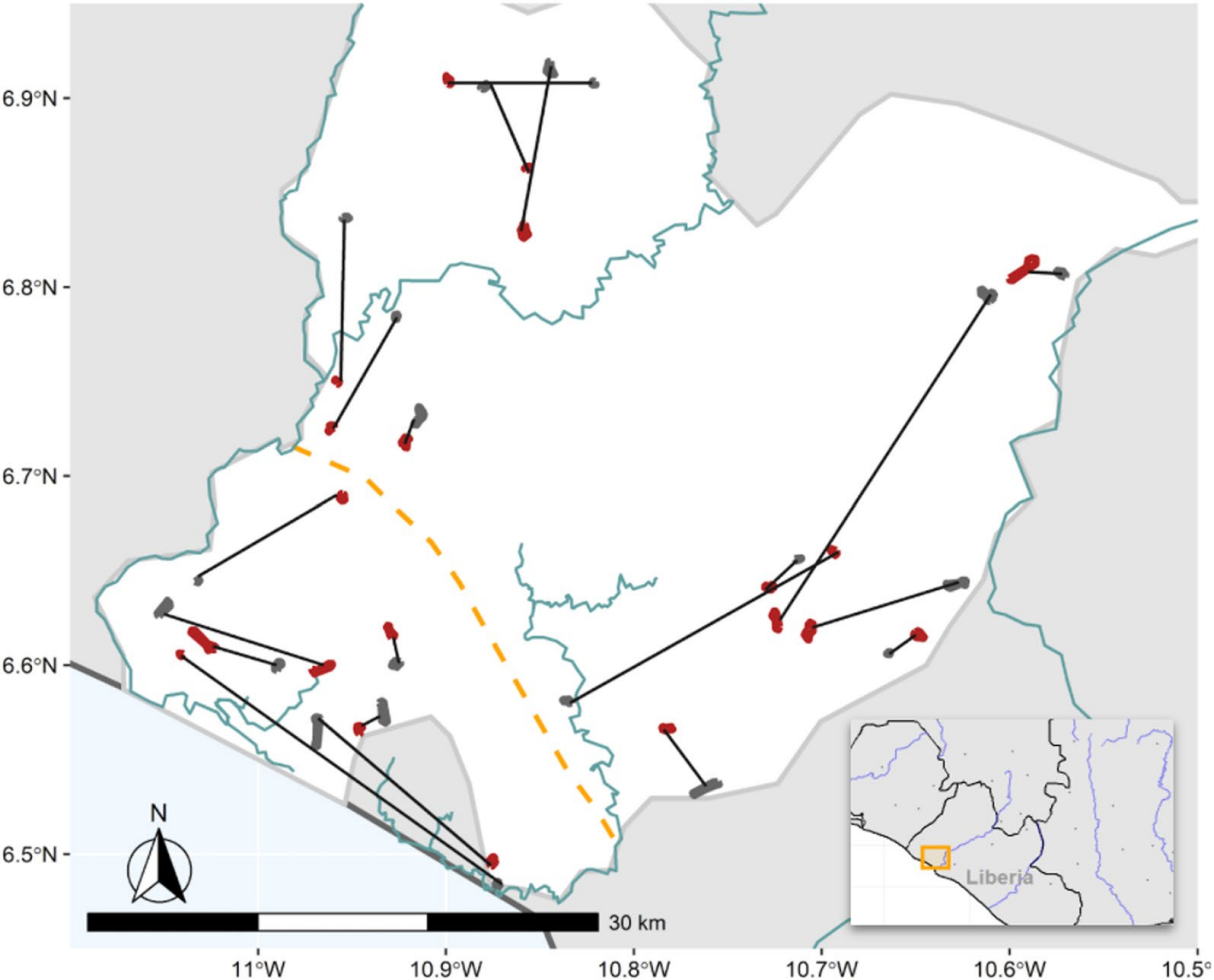
Map of the study area in Bomi County, Liberia. Control clusters are shown in grey, Active clusters are shown in red, pairing is shown with black lines. The division between upland and coastal regions is shown with dashed orange line. Administrative boundaries were used to select villages; no clusters were selected in the central administrative district. Inset: map of region, main map area shown as an orange rectangle

Selected clusters (Fig. 2, Table 1) were randomly assigned to receive DWL in addition to whatever LLINs were already being used, or to act as controls, where no intervention was provided other than the LLINs already in use.

**Table 1:**
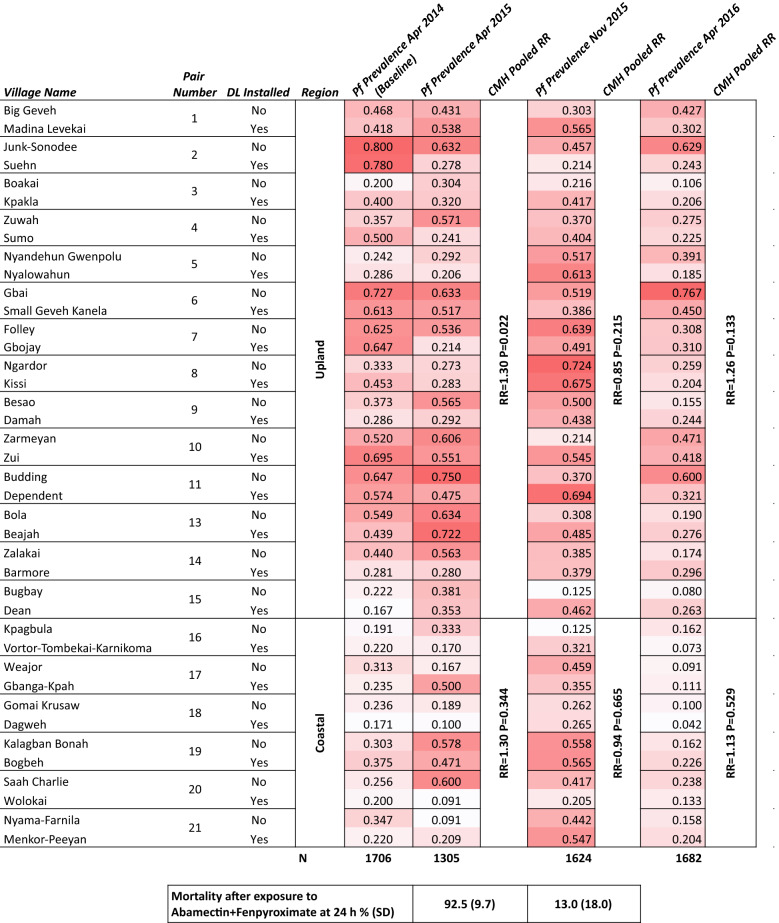
Epidemiological effect of DWL in 20 paired clusters Bomi County, Liberia. Paired clusters with their corresponding village names and cluster numbers are shown along with whether they were randomized to receive DWL or not

DWL was manufactured as a custom prototype by Vestergaard Frandsen SA. This prototype was a non-woven polypropylene impregnated with fenpyroximate and abamectin which are chemicals previously used in horticulture, but have not been applied to mosquito control. The non-woven material had no additional surface treatments. Fenpyroximate is a NADH-coenzyme Q reductase inhibitor (IRAC 21a), while abamectin acts on the glutamate-gated chloride channel (IRAC 6). Chemicals with these modes of action have not been widely used to control malaria mosquitoes.

**Fig. 3:**
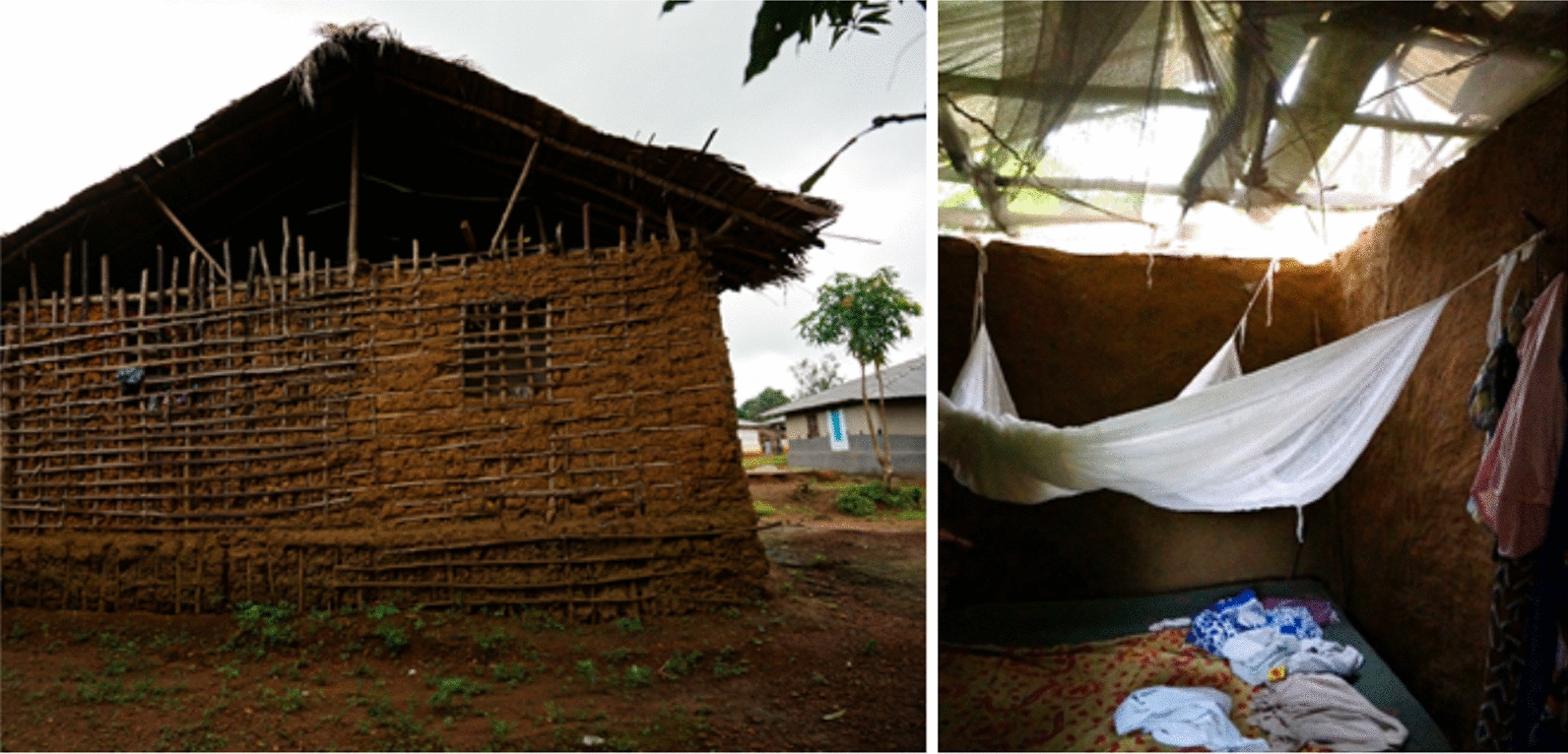
Typical housing design in Bomi County, Liberia showing open gables and ceilings. Exterior photo shows the large gable allowing for cross-breeze and mosquito entry. Interior photo shows interior dividing walls extending approximately 2 m above the floor, an LLIN hung above the bed, and recycled LLINs sewn together to make a ceiling

**Fig. 4:**
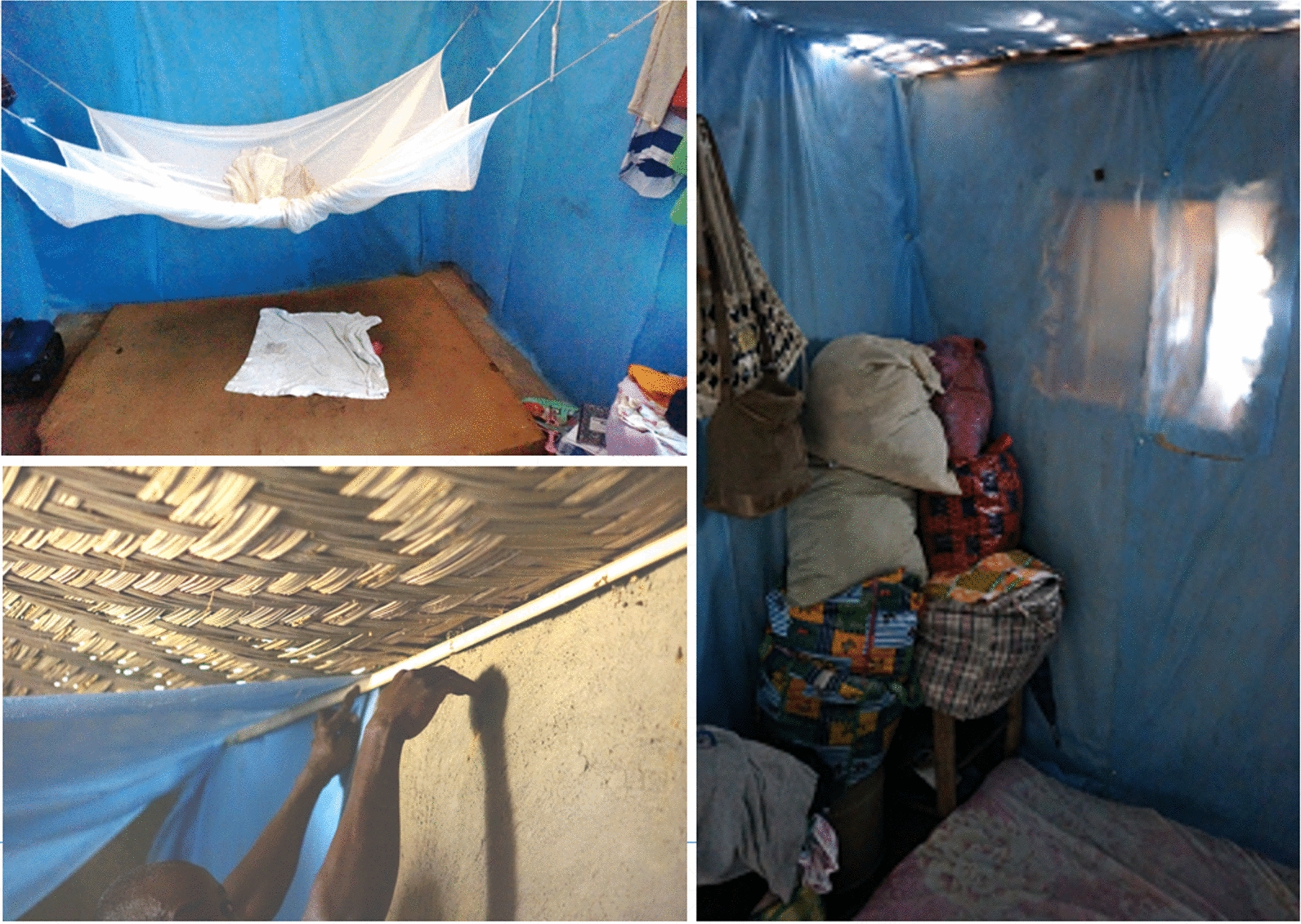
Photos of typical installation of DWL in houses in Bomi County, Liberia. Deltamethrin LLINs were present as an additional control measure in both arms of the study. Ceilings and windows were covered with DWL using wooden strips

Installation of the DWL was conducted by groups of 10 to 12 installers who were selected by village leadership and who were provided with non-cash incentives during the installation period (Additional file 1: Table S1: Installation progress in clusters receiving DWL in Bomi County, Liberia as of December 10, 2014). Houses in which DWL was installed, shared similar structural characteristics and were built out of local material (See Figs. 3 and 4). Personal protective equipment and meals were provided by the study until installation was complete.

Community consultations with village leadership were conducted before study activities commenced and continued throughout the study period. Verbal and written consent was obtained before participation in the survey, including entry of houses for DWL installation and epidemiological surveys. Consent forms were written in simple English, which is the trade language in the area, and village leadership were involved in coordination and translation of the consent process. Treatment or testing for malaria was not conditional on participating in the trial.

## Outcomes

### Outcome measures

Comparison of epidemiological effectiveness between study arms was to be based in the first instance on prevalence of infection with *P. falciparum* in children 2 months to 59 months of age, determined by cross-sectional household malaria indicator surveys conducted in all clusters every 6 months, for a total of two years post-installation (base line). Surface bioavailability and entomological effectiveness of the new active ingredients in DWL through the study period was measured as a secondary outcome.

### Epidemiological outcomes

In April 2014, a baseline epidemiological survey of children under 5 years of age was conducted in 40 clusters. Age, sex, tympanic temperature were recorded and children were tested for *P. falciparum* infection with SD Bioline *Pf* rapid diagnostic test (Abbott Rapid Diagnostics, Gyeonggi-do, Korea). All positive cases were treated with artesunate amodiaquine (Sanofi, Paris, France) following a positive test. The epidemiological survey was repeated after DWL installation at 12, 18 and 24 months after baseline until study completion in April 2016. All children 2 months to 59 months were recruited regardless of whether they participated in earlier surveys. Surveys at 12, 18 and 24 months following baseline were conducted during Ebola virus disease transmission and were subject to increased infection control protocols. This meant that febrile (by infrared thermometer) or other symptomatic children were excluded from the study. Between monitoring periods the NGO continued to support healthcare throughout the region.

### Insecticide bioefficacy and entomological effectiveness outcome

To determine the baseline resistance profile in wild-caught mosquitoes, WHO susceptibility testing was conducted following WHO insecticide guidelines [18]. Bioefficacy of the DWL material against *An. gambiae s.l.* mosquitoes was determined by collecting larvae from three sites in Bomi County: Bahai Town (6°42′43.2"N 10°58′30.0"W), Gbojay (6°39′31.5"N 10°41′36.5"W) and Snowe (6°50′01.6"N 10°49′34.1"W). Larvae were reared to adults and females were tested in WHO cone bioassays modified from WHOPES LLIN guidelines [18]. Samples with dimensions of 30 × 30 cm of DWL from consenting households were taken at 2 m above the floor and the resulting holes were patched. Female mosquitoes aged 2–5 days post eclosion were exposed at a 45° angle for 30 min with 10 mosquitoes per cone and placed in holding cups for 72 h. Due to the variability in assay conditions and wild-caught mosquitoes, the allowable control mortality at 72 h was extended to 20%. The mean mortality was calculated for 12 samples collected at time of installation and 129 samples collected 12 months post installation (Additional file 2: Table S2: Insecticide resistance status of adult mosquitoes exposed to WHO insecticide papers in tube test).

### Sample size

The sample size calculation is based on the principles of cluster randomised trials described by Hayes and Moulton [19]. Sample size was calculated using the n4props function in the R package CRTSize (available at https://rdrr.io/cran/CRTSize), with *P. falciparum* positivity set to 30 and 40%, in experimental and control arms, respectively, number of children per cluster set to 50, ICC = 0.03, alpha = 0.05 and power = 0.8. The trial is intended to show a difference between DWL plus residual LLINs and residual LLINs alone and power calculations are based on prevalence in the DWL arm being significantly less than in the control arm.

## Randomization

### Sequence generation

To assign clusters into experimental and control arms of the study, clusters were paired based on the following covariates: malaria prevalence, population size, LLIN usage and district. Assignment into cluster pairs was conducted using the nbp Matching package in R (code available at https://github.com/couthcommander/nbpMatching). Following pairing, one cluster in each pair was randomly assigned to a study arm by generating pseudo random numbers. The field study coordinator determined the randomization sequence. The field study co-ordinator ran the analysis and assigned the random sequence, enrolled clusters, and assigned clusters to interventions. Consent was sought from village community leaders for inclusion as clusters in the study. Due to security, supply and access constraints, one pair was removed from the study. However, DWL was later separately also installed in the two non-study villages, at the request of the village leadership.

### Statistical methods

To measure the effect of DWL installation on malaria infections, a matched pair design was used, with pairs of village clusters matched within three subregions in Bomi County. There was no weighting by population size. Differences in malaria prevalence between the coastal region and the two upland regions led us to separate the analysis into two segments by region (Table 1). The proportion of *P. falciparum* infected children under 5 years of age in each cluster was calculated. The pooled risk ratio (RR) of *P. falciparum* infections was calculated in SAS (Cary, NC, USA) using the Cochran–Mantel–Haenszel (CMH) test for cluster pairs in each region within Bomi County (upland or coastal).

Mosquito mortality was calculated by scoring dead and alive mosquitoes at 24 and 72 h post exposure to DWL samples and adjusting with Abbot’s formula as required [18]. All other figures and statistics were produced in R software V4.1.2 [20].

## Results

A cluster randomized controlled trial with matched pairing (Fig. 2) was conducted to measure the effect of DWL on *P*. *falciparum* malaria in children under 5. Baseline was April 2014, with follow up outcome monitoring time-points in April 2015, November 2015 and April 2016.

**Fig. 5:**
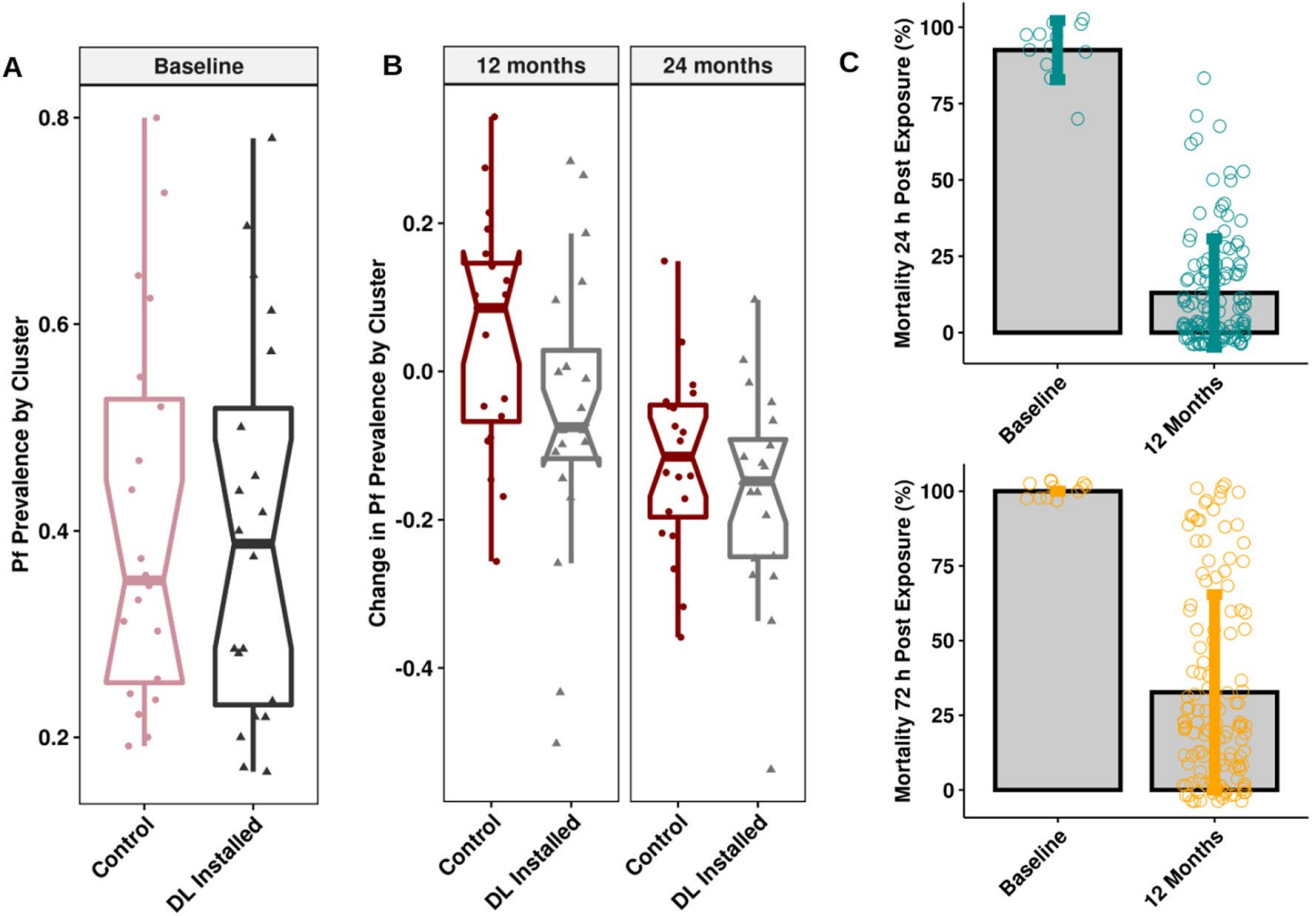
*P. falciparum* prevalence is balanced at baseline, then decreases in both arms during the study period. Bioavailability of insecticide in DWL decreases rapidly. **A** baseline balance in *P. falciparum* (Pf) prevalence between control and experimental (DWL installed) study arms in village clusters in Bomi County, Liberia. Boxplot represents mean, interquartile range, whiskers represent range, notch represents 95% confidence interval. **B** Change in proportion of *P. falciparum* (Pf) prevalence from baseline in April 2014 to April 2015 (12 months), and from baseline to the end of the study in April 2016 (24 months), in each cluster by study arm. Boxplot represents mean, interquartile range, whiskers represent range, notch represents 95% confidence interval. **C** Mortality of *An. gambiae s.l.* following exposure to fenpyroximate abamectin treated DWL, observed at 24 and 72 h post exposure in WHO cone bioassays. Bars represent mean of replicates, dots represent individual bioassays, error bars represent standard deviation. There were no important harms of unintended effects in any of the paired clusters

Installation of DWL resulted in a significant reduction of *P. falciparum* malaria prevalence 12 months later (Table 1) in the 28 Upland clusters of the study (RR = 1.3, p = 0.022). However, this effect was not seen in the 12 Coastal clusters at 12 months (RR = 1.3, p = 0.344). When calculated across all clusters *P. falciparum* prevalence in intervention clusters after 12 months was 34.6% compared to 40.1% in control clusters (p = 0.052). A difference between study arms was not observed at 18 or 24 months following the baseline survey, and reduction in control effect coincided with a significant reduction in bioavailability of insecticides on the DWL (Fig. 5C) after 12 months.

At baseline, the two study arms had similar parasitaemia (Fig. 5A), but as the study progressed, malaria prevalence dropped in both arms (Fig. 5B). The pooled risk ratio (RR) of *P. falciparum* infections was calculated in SAS (Cary, NC, USA) using the Cochran–Mantel–Haenszel (CMH) test for cluster pairs in each region within Bomi County (Upland or Coastal).

Species diversity estimates and *kdr* genotyping revealed that insecticide resistance in the predominant malaria vectors had not changed from the last reports in the area (Additional file 2: Table S2) [9].

## Discussion

Despite decades of mosquito control in Liberia using LLINs and IRS, malaria prevalence remains high. The current study demonstrates partial efficacy of DWL, an approach to mosquito control that is tailored to a typical Liberian house design and is a hybrid of LLINs and IRS. By covering ceilings and windows, the insecticidal material reduced mosquito access to indoor sleeping areas. Installing DWL as a whole house improvement is a promising approach that may provide an effective and appropriate mosquito control measure where LLIN usage is low. Measuring the effect of vector control interventions against malaria is challenging, due to the complexity of the movement of vectors and humans. Modification of housing is especially challenging as uptake is incomplete, and insecticidal materials can be repurposed or modified in unexpected ways. In the current study, an outbreak of Ebola virus disease occurred, which further complicated the installation of DWL and the measurement of its effect.

Mosquito control interventions that improve the whole house have been studied in a variety of contexts, but inconsistencies in experimental design and materials make it challenging to define a best practice for deployment of whole-house insecticidal materials. One theme that does emerge from the limited available literature is that covering eaves and other entry points is an important consideration. Disaster relief agencies have tested ITPS in Sierra Leone [8] finding that delivery of insecticides on the ceiling and walls of tents resulted in reduced malaria transmission. Hut trials in Tanzania [21] found that both pyrethroid and non-pyrethroid DWL performed poorly against resistant malaria vectors. In these studies, the lack of coverage of eaves was cited as a reason for the persistence of indoor biting. Variation in product formulation may further explain some of the differences in results between these studies and the current results.

Feasibility of DWL as an intervention has been analysed, with various approaches aimed at reducing cost and maximizing acceptability. Recent research into the utility of eave tubes is very encouraging [15, 16]. Eave tubes dramatically reduce the surface area of the insecticidal material, but require significant modifications to the structure of the house. In many rural Liberian homes, the large open gables (Fig. 3) likely makes the installation of eave tubes very challenging. Ceiling and eave coverage with DWL is expected to be associated with higher acceptability. Adaptation of a range of vector control interventions to various local contexts is needed, as are comparisons of cost and acceptability of DWL to eave tubes and other housing improvement.

Study findings indicate a significant effect on malaria prevalence at 12 months post baseline, which was not observed in trials of the most comparable DWL prototypes. DWL was observed to be an effective malaria control product in the Upland (inland) region of Bomi County, but less so in the Coastal region. Differences in effect may be due to more stable malaria transmission characteristics inland, compared to the coastal region. Human behaviour characteristics may also have played a role in reducing malaria control effect in the Coastal region, where families may be engaged in outdoor activities as night, related to fishing. This requires further investigation.

Similar prototypes made by the same supplier have been tested in other trials [4, 5, 21], but variation between final finishing and production settings may differ from the one reported here.

Mosquito genotyping revealed pyrethroid resistance at similar levels to that reported in 2012 [9]. Entomological efficacy of the DWL was near 100% at 12 months post installation, although mortality was delayed. The significant drop in bioavailability of the two active ingredients in DWL after 12 months usage, is responsible for the drop in malaria control effect, not resistance to these novel compounds. It is unlikely that either cross resistance is an issue or that a de novo mechanism evolved in this timeframe. Sublethal effects in *Anopheles* with the avermectin class of pesticides, that includes abamectin, have been described previously [22], which may have reduced mosquito lifespans sufficiently to influence transmission. The implications of sublethal effects require further investigation.

Coastal villages compared to Upland (inland) villages showed a dramatic drop in overall malaria prevalence (Table 1, Fig. 5). While DWL may have been responsible for this effect, additional factors, including ivermectin mass drug administration, and a national LLIN distribution in 2015 may have reached coastal villages more readily than inland villages. It is also notable that Ebola screening procedures excluded participants in surveys after 12 months. Severe cases were not included in the study, and their treatment-seeking behaviour is unknown.

Studies in Mozambique with comparable whole house coverage showed marked decrease in indoor biting [14]. Indoor biting was reduced and malaria infections were reduced in migrant worker camps in India where the entire shelter was made from ITPS [23]. Reductions in malaria infection were also observed in temporary shelters in Sierra Leone where walls and ceilings were insecticide treated [8]. Other house improvement approaches currently being tested include eave barrier tubes [15] and screening [24], with the latter showing a measurable effect on anaemia, even with non-insecticidal screening. The results of the current study appear to confirm the need to cover eaves and gables when deploying insecticidal materials similar to DWL. There is a compelling need for further research into the role insecticidal materials can play as housing improvements to achieve scalable means of malaria control in regions where pyrethroid resistance threatens the progress that has been made.

Combining insecticide classes by layering a non-pyrethroid wall liner on top of existing LLINs has been studied previously [6], but in contrast, the current results suggest an additive effect of the two control methods. The displacement of LLIN usage by DWL usage is problematic, however, and has been described elsewhere [2]. Usage of LLINs was not controlled in this study and many observed to forgo LLIN hanging after DWL installation. Although the efficacy of pyrethroid LLINs may be reduced in Liberia [17], the observed decrease in malaria—despite lack of control over LLIN usage between study arms—suggests that DWL as a single intervention is partially effective at decreasing transmission.

It is important to highlight that all children that were followed up had been previously cleared of infection by ASAQ treatment [25]. A drop in prevalence over the 24-month study period occured, and the majority of infections measured after baseline are assumed to be the result of re-infection following treatment, or new infections post weaning.

As a hybrid approach that benefits from aspects of successful use of LLINs and IRS, whole-house deployment of DWL is shown here to be partially effective at decreasing malaria in Liberia, despite an unexpected decline in bioefficacy. The decision to cover gables and eaves resulted from community input, an essential component when developing vector control tools [26, 27], and this likely contributed to a reduction in malaria in DWL protected houses, even after bioavailability declined. Further research into the mechanisms of decreased house entry and insecticide-mediated mortality of indoor biting mosquitoes is needed.

## Limitations

The trial implementation period coincided with the largest Ebola epidemic in recorded history, affecting much of Liberia, neighbouring countries and some more distant countries connected by air travel. The main limitation of this, was that planned outcome monitoring in November 2015 was blocked due to Ebola lock down regulations at that time point. Results of follow on time points indicate that November 2015 would have been the time when DWL insecticide efficacy would have been at its maximum, and a stronger effect may have been seen than at 12 months (April 2015). Bomi County is a post-conflict setting with range of housing styles and a high degree of community disruption in the past decade. Housing included row-house style camps built in rubber and palm oil plantations as well as remote villages with more traditional housing. Pairing and randomization did not account for these differences. No other major limitations occurred across the 20 pairs of clusters.

## Generalisability

DWL is an extremely customisable intervention that can be adapted to different housing types common to rural and urban settings. It can be installed onto any surface material, and can cover window and eve openings, gaps and holes in wall and roof/ceiling structures, which otherwise insects would fly through, as required by individual houses, making the results of the trial highly generalisable to different countries and housing types.

## Conclusions

This trial tested the first DWL product combining two novel none-pyrethroid active ingredients, during a major Ebola epidemic in Liberia. DWL is a promising housing improvement material for the control of malaria mosquitoes, especially where houses feature large open gables and eaves and DWL is installed as an insecticidal barrier to entry. If future generations of DWL have longer lasting residual active ingredient effects, then this product may become a useful additional tool in the IVM toolbox for malaria control.

## Supplementary Information

**Additional file 1: Table S1**. Installation progress in clusters receiving DWLin Bomi County, Liberia as of December 10, 2014. **Fig. S1.** Flowchart of study design. Participants were recruited in 42 clusters for the baseline epidemiological survey. Clusters were matched based on *P. falciparum* prevalence, population size, LLIN usage and district. Surveys were conducted every 6 months, with the exception of 6 months after baseline due to Ebola virus disease restrictions. Weekly case counts in Liberia are shown as a colour gradient in red. Rainfall amounts are shown as a colour gradient in blue, allowing for the visualization of rainy season.

**Additional file 2: Table S2.** Insecticide resistance status of adult mosquitoes exposed to WHO insecticide papers in tube test.

## Data Availability

The trial protocol, together with the datasets used and/or analysed during the current study are available from the corresponding author upon reasonable request.

## Abbreviations

DWL: Durable, insecticidal wall liners
LLIN: Long-lasting insecticidal nets
IRS: Indoor residual spraying
ITPS: Insecticide treated plastic sheeting
