# Supplementary material for: Durable wall lining for malaria control in Liberia: results of a cluster randomized trial
Source: Malar J. 2023 Jan 12;22:15. doi: 10.1186/s12936-022-04429-7 (PMC9837910; doi:10.1186/s12936-022-04429-7)
Supplement: Supplementary file 1 — Additional file 1: Table S1. Installation progress in clusters receiving DWLin Bomi County, Liberia as of December 10, 2014. Fig. S1. Flowchart of study design. Participants were recruited in 42 clusters for the baseline epidemiological survey. Clusters were matched based on P. falciparum prevalence, population size, LLIN usage and district. Surveys were conducted every 6 months, with the exception of 6 months after baseline due to Ebola virus disease restrictions. Weekly case counts in Liberia are shown as a colour gradient in red. Rainfall amounts are shown as a colour gradient in blue, allowing for the visualization of rainy season. [file 12936_2022_4429_MOESM1_ESM.docx]

**Additional Files**

Table S1: Installation progress in clusters receiving DWLin Bomi County, Liberia as of December 10, 2014.

| **Name** | **Month Installation Begun (2014)** | **Total Rooms** | **Rooms Refused** | **DWL RoomCoverage on Dec 10, 2014** | **Population** | **LLIN Use** | **Under 5s Tested at Baseline** | **Pf prevalence** | **Elevation (m above sea level)** |
| --- | --- | --- | --- | --- | --- | --- | --- | --- | --- |
| Barmore | August | 101 | 23 | 77% | 322 | 78% | 32 | 28% | 76 |
| Beajah | September | 200 | 15 | 93% | 310 | 68% | 57 | 44% | 11 |
| Bogbeh | June | 185 | 30 | 84% | 1535 | 81% | 32 | 38% | 41 |
| Dagweh | June | 182 | 27 | 85% | 650 | 39% | 41 | 17% | 33 |
| Damah | July | 139 | 66 | 53% | 608 | 49% | 56 | 29% | 39 |
| Dean | September | 67 | 32 | 52% | 138 | 78% | 18 | 17% | 52 |
| Dependent | July | 167 | 15 | 91% | 325 | 30% | 61 | 57% | 36 |
| Gbanga-Kpah | June | 117 | 10 | 91% | 453 | 85% | 34 | 24% | 52 |
| Gbojay | July | 260 | 81 | 69% | 1500 | 49% | 51 | 65% | 20 |
| Kissi | August | 168 | 22 | 87% | 1680 | 28% | 53 | 45% | 25 |
| Kpakla | June | 105 | 10 | 90% | 330 | 20% | 40 | 40% | 70 |
| Madina-Levekai | June | 201 | 24 | 88% | 610 | 30% | 59 | 42% | 52 |
| Menkor-Peeyan | July | 102 | 22 | 78% | 598 | 59% | 41 | 22% | 23 |
| Nyalowahun | June | 78 | 4 | 95% | 948 | 31% | 28 | 29% | 27 |
| Small Geveh | July | 135 | 20 | 85% | 350 | 71% | 31 | 61% | 120 |
| Suehn | June | 348 | 41 | 88% | 250 | 66% | 50 | 78% | 43 |
| Sumo | June | 200 | 28 | 86% | 765 | 67% | 38 | 50% | 47 |
| Vortor-Tombekai | June | 106 | 39 | 63% | 363 | 94% | 50 | 22% | 20 |
| Wolokai | August | 76 | 15 | 80% | 233 | 94% | 35 | 20% | 21 |
| Zui-Mahe | July | 173 | 18 | 90% | 764 | 81% | 59 | 69% | 33 |
| **Total** |  | **3110** | **542** |  | **12732** |  |  |  |  |
| **Average** |  |  |  | **81%** |  | **60%** | **43.3** | **40%** | **42.05** |


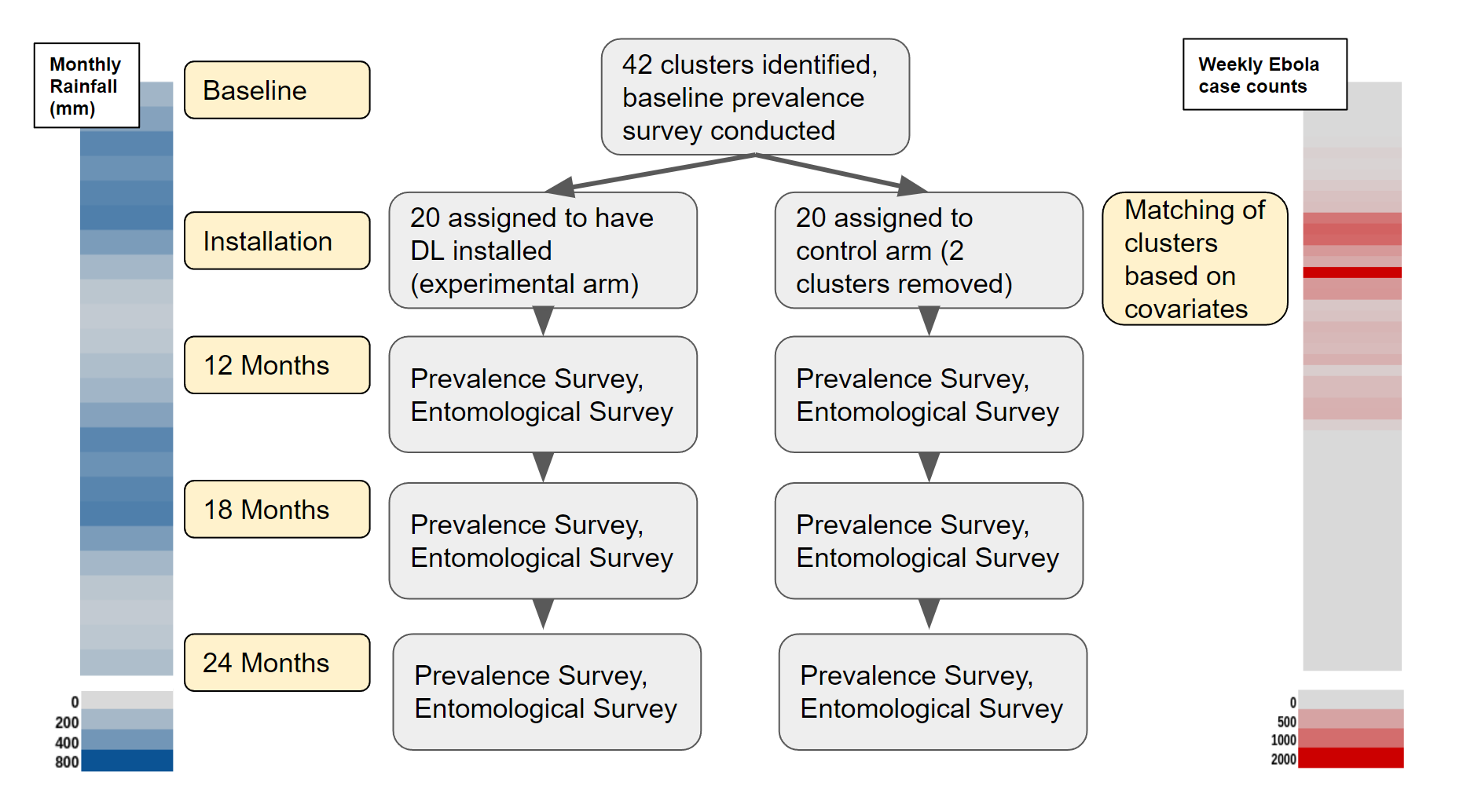


Additional Fig. 1: Flowchart of study design. Participants were recruited in 42 clusters for the baseline epidemiological survey. Clusters were matched based on *P. falciparum* prevalence, population size, LLIN usage and district. Surveys were conducted every 6 months, with the exception of 6 months after baseline due to Ebola virus disease restrictions. Weekly case counts in Liberia are shown as a colour gradient in red. Rainfall amounts are shown as a colour gradient in blue, allowing for the visualization of rainy season.
