# Supplementary material for: Durable wall lining for malaria control in Liberia: results of a cluster randomized trial
Source: Malar J. 2023 Jan 12;22:15. doi: 10.1186/s12936-022-04429-7 (PMC9837910; doi:10.1186/s12936-022-04429-7)
Supplement: Supplementary file 2 — Additional file 2: Table S2. Insecticide resistance status of adult mosquitoes exposed to WHO insecticide papers in tube test. [file 12936_2022_4429_MOESM2_ESM.docx]

Additional File 2

Table S2: Insecticide resistance status of adult mosquitoes exposed to WHO insecticide papers in tube test.

| **Sampling site** | **Insecticide** | **Concen-tration**  **(g/m^3^)** | **KD 60** | **Control KD 60** | **Final test mortality (24 hours)** | **Control % mortality** | **Abbott's Adj. Needed** | **Susceptibility status** |
| --- | --- | --- | --- | --- | --- | --- | --- | --- |
| Gbah | Deltamethrin + PBO | 0.05 | 17 | 0 | 48 | 0 | NO | RESISTANT |
| Gbah | Deltamethrin | 0.05 | 5 | 0 | 6 | 0 | NO | RESISTANT |
| Gbah | DDT | 4 | 0 | 0 | 7 | 0 | NO | RESISTANT |
| Gbah | Bendiocarb | 0.1 | 0 | 0 | 100 | 0 | NO | SUSCEPTIBLE |
| Gbah | Propoxur | 0.1 | 100 | 0 | 100 | 0 | NO | SUSCEPTIBLE |
| Gbah | Fenitrothion | 1 | 18 | 0 | 85 | 0 | NO | RESISTANT |
| Snowe Farm | Bendiocarb | 0.1 | 0 | 0 | 100 | 0 | NO | SUSCEPTIBLE |
| Snowe Farm | Deltamethrin | 0.05 | 2.5 | 0 | 10 | 0 | NO | RESISTANT |
| Snowe Farm | Deltamethrin + PBO | 0.05 | 23 | 0 | 94 | 10 | YES | SUSPECTED RESISTANT |
| Snow Farm | Fenitrothion | 1 | 31 | 0 | 100 | 0 | NO | SUSCEPTIBLE |
| Snowe farm | DDT | 4 | 0 | 0 | 16 | 0 | NO | RESISTANT |
| Gbojay | Bendiocarb | 0.1 | 100 | 0 | 100 | 0 | NO | SUSCEPTIBLE |
| Gbojay | DDT | 0.04 | 0 | 0 | 23 | 0 | NO | RESISTANT |
| Gbojay | Deltamethrin | 0.05 | 0 | 0 | 16 | 0 | NO | RESISTANT |
| Gbojay | Fenitrothion | 0.1 | 1.25 | 0 | 99 | 0 | NO | SUSCEPTIBLE |
| Gbojay | Deltamethrin + PBO | 0.05 | 32.5 | 0 | 89 | 0 | NO | RESISTANT |
